# The Cracking of Teeth in Soldering

**Published:** 1894-07

**Authors:** L. P. Heskell

**Affiliations:** Chicago.


ARTICLE II.

### THE CRACKING OF TEETH IN SOLDERING.

L. P. HESKELL, D. D. S., CHICAGO.

In my own experience, and taking no pains to avoid it, I do not remember the time when I ever had a tooth crack.

Perhaps a few suggestions may not be amiss. If dentists would consider that "cross" pin teeth are more apt to crack than the perpendicular or "straight" pin teeth, they would use fewer of them. They are not only more liable to crack in soldering, but are not as strong for wear. This fact is so apparent it has been a great wonder to me that at the dental depots, when inquiring for "straight" pins and wondering there were so few, I am told that "the dentists all ask for 'cross pins.'" Use less of them, gentlemen, and you will have fewer cracks in soldering and less breakage in wear.

Do not rivet your pins, as the solder flows only on the head of the pin; split the pin, and if the hole is larger than the pin, so much the better, for the solder flows down inside and holds the pin stronger.

Never heat up the case till the borax and solder have been applied. Heat up over the large burner, slowly at first, and then turning on the full head; take about one-half hour. The heat should (in a plate) be thrown by a blow-pipe on the plate first, so that it may be as hot as the backings, as they will easily heat up from their exposed position. Then throw the heat directly on the solder. If necessary to apply more solder do so, but not more borax, as there is danger of cracking the teeth.

Many dentists experience much difficulty in soldering by using the miserable little jewelers' blow-pipes which are not at all suitable for dentists' use, there not being a sufficient volume of flame, and the mouth aperture being so small it has to be taken inside the lips, thereby causing the muscles to become tired. To remedy this I induced the dental goods manufacturers to make a larger blow-pipe—the mouth-piece to be pressed against the lips—which makes soldering much easer.

Another difficulty is found in not having a suitable soldering burner. Formerly I wound fine binding wire over the end of the gas-pipe so as to break the force of the gas jet. But I am now using a burner made for the purpose, which some have called the "Haskell burner," Many use the automatic blow-pipe, but I have found that beginners succeed better with the blow-pipe I have described, and for my own use I far prefer it.—*Dental Review.*

---

ARTICLE III.

